# Loot

**Published:** 1870-07

**Authors:** 


### Loot.

### *Intestinal Puncture in Tympanitis.*

Under the advice of Dr. Fonssagrives, intestinal puncture, as a last resource, has been several times practiced at Toulouse, on two patients suffering with tympanitis. In the first case, the abdomen formed an immense mass, the patient was perfectly cyanosed and suffocating. An exploring trocar was inserted into the most distended part of the lower umbilical region. The gas escaped so violently as to extinguish a candle. The distention returning the next day, two fresh punctures were made in different places, and gave so much relief that the life of the patient was prolonged four days. In another case six punctures were successfully made until the gases were naturally evacuated and the patient cured.—*L' Un Med.—West. Jour. of Med.*

### *M. Auzias Turenne on Rabies.*

This ingenious investigator endeavors to establish, (in a paper read before the Academy of Medicine of Paris,) a parallel between the phenomena of rabies and those of syphilis. It will be remembered that Marochetti considered that the virus, after having been absorbed by the wound, enters the circulation, and then gathers under the tongue. From the third to the ninth day small vesicles appear on either side of the frenum linguæ; they contain the virus, and are called lyssæ. Marochetti believed that by cauterizing these vesicles the further progress of the disease would be prevented. Experience has not confirmed his views; but M. Turenne, starting from these phenomena, has attempted to liken rabies to syphilis, and lyssæ to the infecting chancre.—*Dominion Med. and Surg. Journal.*

### *New Remedies for Burns.*

Two new remedies for burns are added to the long list. The first is charcoal. A piece of vegetable charcoal laid on a burn at once soothes the pain, says the *Gazette Medicale*, and if kept applied for an hour cures it completely. The second one is sulphate of iron. This was tried by M. Joel, in the Children's Hospital, Lausanne. In this case, a child, four years of age, had been extensively burnt, suppuration was abundant and so offensive that the ward was uninhabitable. M. Joel ordered the child a tepid bath, containing a couple of pinches of sulphate of iron. This gave immediate relief to the pain, and being repeated twice a day—

twenty minutes each bath—the suppuration decreased, lost its odor, and the child was soon convalescent.—*Med. Press and Circular*, *April* 6, 1870.—*Med. News.*

Three parts of any simple oil, linseed, whale, or olive, with one part of Tilden's Solution of Carbolic Acid, has afforded us most excellent results in severe burns, from kerosene explosions. But the simplest plan, because the materials are everywhere easily obtained, is to melt together one part of beeswax and two parts of fresh lard, dip cloths in this and apply accurately to the surface. These will thoroughly exclude the air, can easily be renewed, and, in short, accomplish all that can be expected from external appliances. This last application relieved a friend of ours from a most frightful powder burn of the face with scarcely a mark left behind except from some particles of powder.

### *Chloral and Chloroform.*

Communications on chloral multiply daily. Among some of its curious effects may be noticed an observation of M. Liégeois at the Sociète de Chirurgie that in a case in which he had performed a minor operation under the influence of chloral, which produced sleep, but not anæsthesia, he resorted to chloroform. To his great surprise he found that the association, so far from increasing the effects, gave rise to excitement which lasted as long as the inhalations were continued. The fact is the more to be remarked as tending to disprove the identity of action between chloral and chloroform insisted upon by Liebreich. M. Giraldés, proceeding in a different direction, administered to infants who have been chloroformed and remained very agitated, a chloral draught. The effect of the association was to produce peaceful sleep for from five to eleven hours. Since then he has frequently employed chloral, either in mixture or enema, whenever children have remained excited after chloroform, and always with success. M. Demarquay observed that he has continued, with advantage, his practice of giving patients immediately after operation successive doses of two or three to five grammes of chloral until sleep is produced. All subjects are, however, far from exhibiting the same effects from the action of chloral. In some, when it is given immediately after the operation, it produces a quiet sleep and deep calm which lasts all day, and prevents any of the pain consequent on the traumatism being felt. Others proved refractory to the action of chloral, which is sometimes rejected by vomiting. As a medium dose he gives two grammes in two spoonsful of syrup diluted with water. M. Giraud-Teulon has observed the same excitement produced by administering chloral in children that have been etherized as related by M. Liégeois in those who have been chloroformed. —*Ibid.*

***Management of Pertussis.***

In Newark, N. J., most of the physicians send their patients to the purifying room of the gas works, to spend an hour or two daily, the inhalation of the fumes arising from the gas passing through lime having the effect of moderating and shortening the disease. In cases where it is inconvenient to visit the works, carbonate of lime has been produced therefrom, and placed in vessels about the room in quantities sufficient to impregnate the atmosphere, and has been attended with good results. When access to gas works cannot be had, the hydro phenil, which, when unpurified, is called benzine, is recommended by Braithwaite; and the same results obtained as from a residence in the purifying chambers of the gas works. Ten or fifteen drops are given in a little water to a child every day, and as soon as it is asleep a few drops are sprinkled on the pillow, so that the smell is diffused throughout the room.—*Trans. of Med. Soc. of New Jersey.*

***Cancrum Oris.***

Abstract of a paper read at a meeting of the Liverpool Medical Association by Thomas R. Glynn, M.B., Lond. (*Liverpool Medical and Surgical Reports and Half-Yearly Abstract*).

The author confined the term to those cases in which gangrene of the lips or cheeks is a prominent feature. He did not think it was of the nature of a specific disease; because,—1st. It never attacks children as a distinct disease preceded by characteristic symptoms, though some have affirmed that it does. 2d. It is always the consequence of some severe illness, especially of the eruptive fevers, and, of these, measles most frequently precedes it. 3d. It is not infectious, though sometimes epidemic, but only as a consequence of the exanthemata. It very rarely attacks several children in a family simultaneously. It appears to depend upon great deterioration of the blood, springing from a general adynamic state, which may originate from many and various depressing causes. It is much more rarely met with among adults than among children, though not unknown among the former. The facts that the mucous membrane of the mouth is more liable to disease in children, and that measles occur chiefly among them, account partly for their great liability to cancrum oris. It has been known to occur at so early an age as nine days. It rarely attacks both cheeks, except as the result of mercurial salivation. Girls are most subject to it, and the majority of cases occur in large towns and manufacturing districts. It is emphatically a disease of the poor. Sometimes symptoms almost like scurvy are present, and the author expressed the opinion that it is not improbable some intimate relation may exist betwen scurvy and cancrum oris. He does not believe that mercury is the most common cause of the disease, as some think. He considered the gangrene to be the result of a low erysipelatous inflammation, commencing in the mucous membrane of the mouth. Gangrene of other parts may occur as complications; also pneumonia, which is very common; pleurisy, pericarditis, and other diseases of the mucous and serous membranes. The rate of mortality in cancrum oris has been excessively high. With regard to treatment, the author remarked that before applying escharotics, the general condition should be considered rather than the local. Nitric acid is useful at first, but its repeated application is injurious. Antiseptics are of great use. Tonics and stimulants constitute the proper internal treatment. The author gave the details of two cases, treated successfully by the use of chlorate of potash wash, and the internal administration of ammonia, chlorate of potash, and bark, with beef tea, wine, and brandy.—*Dental Cosmos.*

***Almost Complete Ossification of the Human Body.***

B., a youth of 17, came under my notice, first, about four years ago with extensive ossification of the muscular system, which continued to progress till his death, which occurred a short time since. The early history of his case, as given by his family, is as follows:

He was a healthy, stout child at the age of eight months, at which time his parents moved to Texas by land. During their continuance on the road and camping out, they first discovered tumors about, in different parts of his body, the size of small marbles, and very hard. These tumors gave, apparently, but little pain, and in no way affected the general health. After an interval of twelve or eighteen months the tumors began to disappear by absorption, and about the same time stiffness of the joints was manifested. Gradual anchylosis followed, and at the age of ten years the hip-joints with those of the arms were completely anchylosed. The ossific matter began then to be deposited in the muscular system. The chest became enclosed with a complete sheet of bone, leaving no traces of outline of the ribs. The head was immovably fixed by the ossification of the sterno-cleido mastoideus on each side. The muscles of mastication were as yet unaffected, and his ability to chew his food not impaired. His digestion was good, and his mental faculties more than ordinary. He could as yet use wrists and hands, his arms being stiffened at right angles, thus allowing him to whittle a little with a pen-knife. When placed upon his feet he could move slowly and cautiously over a smooth surface, but if he got a fall, was unable to rise unassisted.

Movement, however, as the anchylosis progressed, became more and more difficult and totally impossible at a later period. He did not increase in stature after his tenth year, and never manifested any signs of virility. The forms of the muscles were perfectly preserved and could be distinctly felt through the skin, which adhered closely to them. The deposition of ossific matter continued during his life, and, when he died he was almost complete bone. Up to the hour of his death his mind continued clear and unimpaired, and his digestion until a short time previous, and he was cheerful and apparently happy.—*W. M. Byers, M. D.*, *New Orleans Journal of Medicine.*

### *Rip Van Winkle, M. D.*

An After Dinner Prescription. Taken by the Massachusetts Medical Society, at their Meeting held May 25th, 1870.

[We are indebted to the *Boston Medical Journal*, for Prof. O. W. Holmes' Poem, which we reproduce because it contains philosophy as well as fun. Comment is unnecessary—" Good wine needs no bush."]

Canto First.

Old Rip Van Winkle had a grandson, Rip,
Of the paternal block a genuine chip;
A lazy, sleepy, curious kind of chap;
He, like his grandsire, took a mighty nap,
Whereof the story I propose to tell
In two brief cantos, if you listen well.

The times were hard when Rip to manhood grew;
They always will be when there's work to do;
He tried at farming—found it rather slow—
And then at teaching—what he didn't know;
Then took to hanging round the tavern bars,
To frequent toddies and long-nine cigars,
Till Dame Van Winkle, out of patience, vexed
With preaching homilies, having for their text
A mop, a broomstick—aught that might avail
To point a moral or adorn a tale,
Exclaimed—" I have it! Now then, Mr. V.!
He's good for *something*—make him an M.D."!

The die was cast; the youngster was content;
They packed his shirts and stockings, and he went.
How hard he studied it were vain to tell—
He drowsed through Wistar, nodded over Bell,
Slept sound with Cooper, snored aloud on Good;
Heard heaps of lectures—doubtless understood—
A constant listener, for he did not fail
To carve his name on every bench and rail.

Months grew to years; at last he counted three,
And Rip Van Winkle found himself M. D.

Illustrious title! in a gilded frame
He set the sheepskin with his Latin name,
RIPUM VAN WINKLUM, QUEM we—SCIMUS—know
IDONEUM ESSE—to do so and so;
He hired an office; soon its walls displayed
His new diploma and his stock in trade,
A mighty arsenal to subdue disease
Of various names, whereof I mention these:
Lancets and bougies, great and little squirt,
Rhubarb and Senna, Snakeroot, Thoroughwort,
Ant. Tart., Vin. Colch., Pil. Cochiæ and Black Drop,
Tinctures of Opium, Gentian, Henbane, Hop,
Pulv. Ipecacuanhæ, which for lack
Of breath to utter men call Ipecac,
Camphor and Kino, Turpentine, Tolu,
Cubebs, "Copeevy," Vitriol—white and blue,
Fennel and Flaxseed, Slippery Elm and Squill,
And roots of Sassafras and "Sassaf'rill,"
Brandy—for colics—Pinkroot, death on worms—
Valerian, calmer of hysteric squirms,
Musk, Assafœtida, the resinous gum
Named from its odor—well, it does smell some—
Jalap, that works not wisely, but too well,
Ten pounds of bark and six of Calomel.

For outward griefs he had an ample store,
Some twenty jars and gallipots, or more:
*Ceratum simplex*—housewives oft compile
The same at home, and call it "wax and ile";
*Unguentum Resinosum*—change its name,
The "drawing salve" of many an ancient dame;
*Argenti Nitras*, also Spanish flies,
Whose virtue makes the water-bladders rise—
(Some say that spread upon a toper's skin
They draw no water, only rum or gin)—
Leeches, sweet vermin! don't they charm the sick?
And Sticking-plaster—how it hates to stick!
*Emplastrum Ferri*—ditto *Picis*, Pitch;
Washes and Powders, Brimstone for the——which,
*Scabies* or *Psora*, is thy chosen name
Since Hahnemann's goosequill scratch'd thee into fame,
Proved thee the source of every nameless ill,
Whose sole specific is a moonshine pill,
Till saucy science, with a quit grin,
Held up the Acarus, crawling on a pin?
—Mountains have labored and have brought forth mice:
The Dutchman's theory hatched a brood of—twice
I've well nigh said them—words unfitting quite
For these fair precincts and for ears polite.

The surest foot may chance at last to slip,
And so at length it proved with Doctor Rip.
One full-sized bottle stood upon the shelf
Which held the medicine that he took himself:
Whate'er the reason, it must be confessed
He filled that bottle oftener than the rest;
What drug it held I don't presume to know—
The gilded label said "Elixir Pro."

One day the Doctor found the bottle full,
And, being thirsty, took a vigorous pull,
Put back the "Elixir" where 'twas always found,
And had old Dobbin saddled and brought round.
—You know those old-time rhubarb-colored nags
That carried Doctors and their saddle-bags;
Sagacious beasts! they stopped at every place
Where blinds were shut—knew every patient's case—
Looked up and thought—the baby's in a fit—
*That* won't last long—he'll soon be through with it;
But shook their heads before the knockered door
Where some old lady told the story o'er
Whose endless stream of tribulation flows
For gastric griefs and peristaltic woes.

What jack o' lantern led him from his way,
And where it led him, it were hard to say;
Enough that wandering many a weary mile
Through paths the mountain sheep trod single file,
O'ercome by feelings such as patients know
Who dose too freely with "Elixir Pro.,"
He tumbl—dismounted, slightly in a heap,
And lay, promiscuous, lapped in balmy sleep.

Night followed night, and day succeeded day,
But snoring still the slumbering Doctor lay.
Poor Dobbin, starving, thought upon his stall,
And straggled homeward, saddle-bags and all;
The village people hunted all around,
But Rip was missing—never could be found.
"Drownded," they guessed;—for more than half a year
The pouts and eels *did* taste uncommon queer;
Some said of apple-brandy—other some
Found a strong flavor of New England rum.

—Why can't a fellow hear the fine things said
About a fellow when a fellow's dead?
The best of doctors—so the press declared—
A public blessing while his life was spared,
True to his country, bounteous to the poor,
In all things temperate, sober, just and pure;
The best of husbands! echoed Mrs. Van,
And set her cap to catch another man.

—So ends this Canto—if it's *quantum suff.*,
We'll just stop here and say we've had enough,
And leave poor Rip to sleep for thirty years;
I grind the organ—if you lend your ears
To hear my second Canto, after that
We'll send around the monkey with the hat.

### Canto Second.

So thirty years had passed—but not a word
In all that time of Rip was ever heard;
The world wagged on—it never does go back—
The widow Van was now the widow Mac—

France was an Empire—Andrew J. was dead,
And Abraham L. was reigning in his stead.
Four murderous years had passed in savage strife,
Yet still the rebel held his bloody knife.
—At last one morning—who forgets the day
When the black cloud of war dissolved away?
The joyous tidings spread o'er land and sea,
Rebellion done for! Grant has captured Lee!
Up every flagstaff sprang the Stars and Stripes—
Out rushed the Extras wild with mammoth types—
Down went the laborer's hod, the schoolboy's book—
"Hooraw!" he cried—"the rebel army's took!"
Ah! what a time! the folks all mad with joy:
Each fond, pale mother thinking of her boy;
Old gray-haired fathers meeting—Have-you-heard?
And then a choke—and not another word;
Sisters all smiling—maidens, not less dear,
In trembling poise between a smile and tear;
Poor Bridget thinking how she'll stuff the plums
In that big cake for Johnny when he comes;
Cripples afoot—rheumatics on the jump,
Old girls so loving they could hug the pump,
Guns going bang! from every fort and ship—
They banged so loud at last they wakened Rip.

I spare the picture, how a man appears
Who's been asleep a score or two of years;
You all have seen it to perfection done
By Joe Van Wink—I mean Rip Jefferson.
Well, so it was—old Rip at last came back,
Claimed his old wife—the present widow Mac—
Had his old sign regilded, and began
To practice physic on the same old plan.

Some weeks went by—it was not long to wait—
And "please to call" grew frequent on the slate.
He had, in fact, an ancient, mildewed air,
A long grey beard, a plenteous lack of hair—
The musty look that always recommends
Your good old Doctor to his ailing friends.
—Talk of your science! after all is said
There's nothing like a bare and shiny head—
Age lends the graces that are sure to please,
Folks want their Doctors mouldy, like their cheese.

So Rip began to look at people's tongues
And thump their briskets (called it "sound their lungs"),
Brushed up his knowledge smartly as he could,
Read in old Cullen and in Doctor Good.
The town was healthy; for a month or two
He gave the sexton little work to do.

About the time when dogday heats begin,
Measles and mumps and mulligrubs set in;
With autumn evenings dysentery came,
And dusky typhoid lit his smouldering flame;
The blacksmith ailed—the carpenter was down,
And half the children sickened in the town.

The sexton's face grew shorter than before—
The sexton's wife a brand-new bonnet wore—
Things looked quite serious—Death had got a grip
On old and young, in spite of Doctor Rip.

And now the Squire was taken with a chill—
Wife gave " hot drops "—at night an Indian pill;
Next morning, feverish—bedtime, getting worse,
Out of his head—began to rave and curse;
The Doctor sent for—double quick he came:
*Ant. Tart. gran. duo*, and repeat the same
If no et cetera. Third day—nothing new;
Percussed his thorax—set him cussing, too—
Lung-fever threatening—something of the sort—
Out with the lancet—let him blood—a quart—
Ten leeches next—then blisters to his side;
Ten grains of calomel——just then he died.

The Deacon next required the Doctor's care—
Took cold by sitting in a draught of air—
Pains in the back, but what the matter is
Not quite so clear—wife calls it " rheumatiz."
Rubs back with flannel—gives him something hot—
"Ah!" says the Deacon, "that goes *nigh* the spot."
Next day a *rigor*—run, my little man,
And say the Deacon sends for Doctor Van.
The Doctor came—percussion as before,
Thumping and banging till his ribs were sore—
" Right side the flattest "—then more vigorous raps—
Fever—that's certain—pleurisy, perhaps.
A quart of blood, will ease the pain, no doubt,
Ten leeches next will help to suck it out,
Then clap a blister on the painful part—
But first two grains of *Antimonium Tart.*
Last, with a dose of cleansing calomel
Unload the portal system——that sounds well!

But when the self-same remedies were tried,
As all the village knew, the Squire had died;
The neighbors hinted—" this will never do,
He's killed the Squire—he'll kill the Deacon too."

—Now when a doctor's patients are perplexed,
A *consultation* comes in order next—
You know what that is? In a certain place
Meet certain doctors to discuss a case
And other matters, such as weather, crops,
Potatoes, pumpkins, lager beer and hops.
For what's the use?—there's little to be said,
Nine times in ten your man's as good as dead—
At best a talk (the secret to disclose)
Where three men guess and *sometimes* one man knows.

The counsel summoned came without delay—
Young Doctor Green and shrewd old Doctor Gray—
They heard the story—" Bleed!" says Doctor Green,
" That's downright murder! cut his throat, you mean!

Leeches! the reptiles! Why, for pity's sake,
Not try an adder or a rattlesnake!
Blisters! Why bless you, they're against the law—
It's rank assault and battery if they draw!
Tartrate of Antimony! shade of Luke,
Stomachs turn pale at thought of such rebuke!
The portal system! What's the man about?
Unload your nonsense! Calomel's played out!
You've been asleep—you'd better sleep away
Till some one calls you"——
"Stop!" says Doctor Gray—
"The story is you slept for thirty years;
With brother Green, I own that it appears
You must have slumbered most amazing sound;
But sleep once more till thirty years come round,
You'll find the lancet in its honored place,
Leeches and blisters rescued from disgrace,
Your drugs redeemed from fashion's passing scorn,
And counted safe to give to babes unborn."

Poor sleepy Rip, M.M.S.S., M.D.,
A puzzled, serious, saddened man was he;
Home from the Deacon's house he plodded slow
And filled one bumper of "Elixir Pro."
"Good bye," he faltered, "Mrs. Van, my dear!
I'm going to sleep, but wake me once a year;
I don't like bleaching in the frost and dew,
I'll take the barn, if all the same to you.
Just once a year—Remember! no mistake!
Cry 'Rip Van Winkle! time for you to wake!'
Watch for the week in May when laylocks blow,
For then the Doctors meet, and I must go."

Just once a year the Doctor's worthy dame
Goes to the barn and shouts her husband's name,
"Come, Rip Van Winkle!" (giving him a shake)
"Rip! Rip Van Winkle! time for you to wake!
Laylocks in blossom! 'tis the month of May—
The Doctors' meeting is this blessed day,
And come what will, you know I heard you swear
You'd never miss it, but be always there!"

And so it is, as every year comes round
Old Rip Van Winkle here is always found.
You'll quickly know him by his mildewed air,
The hayseed sprinkled through his scanty hair,
The lichens growing on his rusty suit—
I've seen a toadstool sprouting on his boot—
—Who says I lie? Does any man presume—
Toadstool? No matter—call it a mushroom.
Where is his seat? He moves it every year;
But look, you'll find him—he is always here—
Perhaps you'll track him by a whiff you know—
A certain flavor of "Elixir Pro."

Now, then, I give you—as you seem to think
We can drink healths without a drop to drink—
Health to the mighty sleeper—long live he!
Our brother Rip, M.M.S.S., M.D.!

